# Alternative Type of the Trail Making Test in Nonnative English-Speakers: The Trail Making Test-Black & White

**DOI:** 10.1371/journal.pone.0089078

**Published:** 2014-02-13

**Authors:** Hyun Jung Kim, Min Jae Baek, SangYun Kim

**Affiliations:** 1 Clinical Neuroscience Institute, Seoul National University Bundang Hospital, Bundang-gu, Seoungnam-si, Kyeonggi-do, Korea; 2 Department of Neurology, Seoul National University Bundang Hospital, Bundang-gu, Seoungnam-si, Kyeonggi-do, Korea; University of Maryland, College Park, United States of America

## Abstract

**Objective:**

The Trail Making Test (TMT) has its limitations when applied to Eastern cultures due to its reliance on the alphabet. We looked for an alternative tool that is reliable and distinguishable like the TMT and devised the Trail Making Test Black & White (TMT-B&W) as a new variant. This study identifies the applicability of the TMT-B&W as a useful neuropsychological tool and determines whether the TMT-B&W could play an equivalent role as the TMT.

**Methods:**

The TMT-B&W uses numbers encircled by black or white circles as stimuli, instead of using the alphabet. A total of 138 participants were including containing groups of 31 cognitively normal controls (NC), 55 mild cognitive impairment (MCI), and 52 people with Alzheimer’s disease (AD). Along with the TMT-B&W, the TMT and other neuropsychological tests were administered to all subjects.

**Results:**

A considerably low dropout rate for TMT B&W demonstrates that all participants were more willingly engaged in the TMT B&W than the TMT. In particular, subjects with cognitive impairments or lower levels of education performed better on the TMT-B&W than the TMT. The difference in time-to-completion of the TMT-B&W was significant according to the level of cognitive impairment. The TMT-B&W revealed a high correlation with the TMT and frontal lobe function test.

**Conclusion:**

The TMT-B&W is as reliable and effective as the TMT. It is worth developing a new variant of the TMT.

## Introduction

The Trail Making Test (TMT) [1] is a widely used neuropsychological test for identifying mild cognitive impairment and mild dementia [2]–[4]. The TMT measures psychomotor speed, attention, sequencing, mental flexibility, and visual scanning [5]–[7].

**Figure 1:**
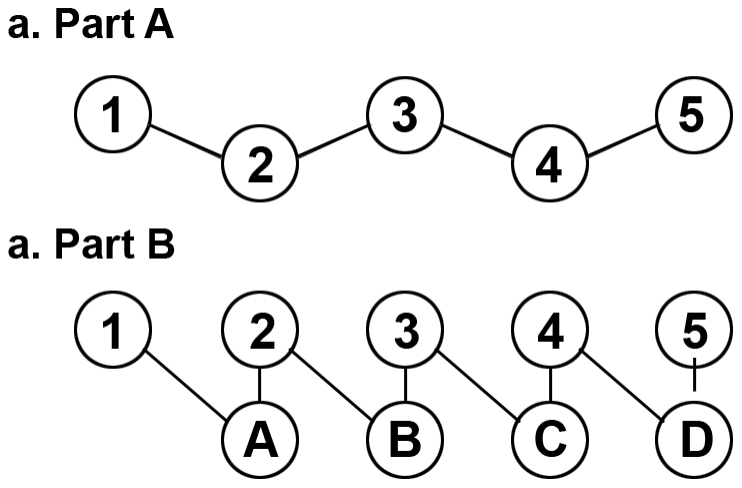
Trail Making Test. Part A consists of the encircled numbers from 1 to 25 randomly distributed on the test sheet. Part B constitutes of the encircled numbers from 1 to 13 and the encircled letters from A to L that are randomly distributed on the test sheet.

The TMT is frequently administered in English-speaking countries with limited utilization in cross-cultural contexts because of the use of the English alphabet on TMT-B [8]. The TMT consists of two parts A and B (TMT-A, TMT-B) which assess the different cognitive processes. TMT-A requires an individual to connect randomly distributed numbers in an ascending order, while on the other hand TMT-B, consists of both numbers and letters, requiring participants to connect numbers and letters alternatively (See Figure 1–a and b). The TMT-A is primarily a test of visual attention skills. It includes perceptual tracking and simple sequencing tasks, whereas the TMT-B, with the additional tasks associated with alternating the sequence pattern, is a test which is used as an index of the frontal executive function [9]–[11]. Therefore, Part B is thought to be a more sensitive measure for cerebral dysfunction than Part A [12].

The effects of age and level of education have been widely suggested to affect the completion time of the TMT. Most studies have shown that subjects who are old and/or with low education take more time to complete the TMT in total, most notably on the TMT-B. The obvious verbal or linguistic component in the alphabetic sequencing aspect of Part B places illiterate and nonnative English-speakers at a distinct disadvantage[13]–[17] which led to a poor performance bias on the TMT-B among Korean seniors, particularly those with a lower education level who could not read English letters. In a clinical domain, a number of studies reported Korean elderly; poorly educated elderly; and patients with mild cognitive impairment often fail to perform well on the TMT-B [18].

The Color Trails Test (CTT) was developed to overcome the shortcomings of the TMT, with the intention of minimizing the cultural bias and offering reliable cognitive measures for diverse populations [19]. However, the CTT was not considered to be similar to the TMT, due to differences in trace and a possible Stroop effect on CTT-B [20]. Moreover, its pink and yellow backgrounds prevent the CTT from being considered an accurate tool for those with color blindness or those with visual defects. In addition, preparing expensive colored paper for the test is burdensome.

For those reasons, we developed the Trail Making Test-Black & White (TMT-B&W). Unlike the CTT, part B of the TMT-B&W demonstrates two sets of numbers (from 1 to 25) in each color(black and white) which requires a subject to connect the numbers in ascending order alternating between the two color sets. We matched the trace of TMT-B&W with that of the TMT. The purpose of this study is: (1) to examine the applicability of the TMT-B&W (2) to evaluate if there is a potential advantage of TMT B&W when it is administered to Eastern populations.

## Methods

### Ethical Issue

This study was approved by the Seoul University Hospital Institutional Review Board of each participating site and written informed consent was obtained from all subjects before all procedures. Participants who declined to participate, or did not participate, were eligible for treatment and were not disadvantaged in any other way by not participating in this study.

The approval number of IRV is “B-1306/208–107”.

### Study Population

This study included 138 outpatients with 31 cognitively normal controls (NC); 55 patients with mild cognitive impairment (MCI); and 52 patients with Alzheimer’s disease (AD), age ranged from 50 to 80. Those who could not read letters and were poorly educated were also included in the sample. Every participant had reportedly experienced some sort of memory impairment before they came to the Clinical Neuroscience Institute of Seoul National University Bundang Hospital. At the baseline visit, a clinician screened subjects and diagnose them into three groups.

We recruited some of the outpatients from a health care center of Seoul National University Bundang Hospital and caregivers who were serving patients undergoing treatment at the Clinical Neuroscience Institute of Seoul National University Bundang Hospital as subjects of the NC group. The criteria for the NC were as follows: 1) no cognitive complaints verified by an informant; 2) higher scores, or at most one standard deviation below, than the mean score of Mini-Mental State Examination (MMSE) for adjusted education and age [21]; 3) absence of significant impairment in any cognitive functions; 4) preserved activities of daily living (ADL) [22]–[23]; 4) no causes of diseases that would undermine cognitive functions [24]; 5) Geriatric Depression Scale (GDS) scored <17 on the 30 item scale in the past one week.

Criteria for diagnosing MCI patients [25], were as follows: 1) cognitive complaints verified by an informant; 2) objectively abnormal cognitive impairment in one or more cognitive functions; 3) preserved ADL; 4) normal visual and auditory functions; 5) no neurological or psychiatric diseases 6) failed to meet the diagnostic criteria of dementia based on the National Institute of Neurological and Communicative Disorders and Stroke and Alzheimer’s Disease and Related Disorders Association (NINCDS-ADRDA) [26].

Among AD patients, 52 were considered to have probable AD with mild dementia severity. All AD patients met the following criteria: 1) being diagnosed as probable AD according to the NINCDS-ADRDA; 2) having mild dementia severity with Clinical Dementia Rating Scale Sum of Box (CDR-SOB) scores between 2.5 and 4.0 [27]–[28]; 3) no impairment in vision and hearing; 4) no diseases related to neurological symptoms or psychological dysfunctions.

The demographic characteristics of the participants are described in Table 1. There was a significant difference of age and scores on MMSE among the groups of NC, MCI, and AD (*F*_(2,135)_ = 23.31, *F*_(2,135)_ = 51.86, *p*<0.05).

**Table 1:** Demographic Data for Participant Groups.

		Age (in years)		Education (in years)		MMSE (scores)	
Group	N	M	SD	M	SD	M	SD
NC	31	62.55* †	6.20	11.61	6.18	28.22* †	1.89
MCI	55	69.11* ‡	7.47	8.45	6.59	25.65* ‡	3.22
AD	52	74.06† ‡	8.06	8.45	6.64	20.09† ‡	4.99

Abbreviations; N, number of participants; M, Mean; SD, Standard deviation; NC, Normal Controls; MCI, Mild Cognitive Impairment; AD, Alzheimer’s disease; MMSE; Mini Mental Status Examination.

Note.

*p<0.05 for NC versus MCI.

† p<0.05 for NC versus AD.

‡ p<0.05 for MCI versus AD.

### Procedure and Materials

After gathering all participants’ personal information through one-on-one interviews, Clinical Dementia Rating (CDR) [29] was also carried out. Each participant was then individually administered the TMT and TMT-B&W and then given a neuropsychological test to measure diverse cognitive functions. Given the fact that pre-exposure to cognitive tests may influence a test subject’ performance, we administered the TMT and TMT-B&W prior to the other tests. Half of the participants tested the TMT-B&W before the TMT.

### Trail Making Test-Black & White

**Figure 2:**
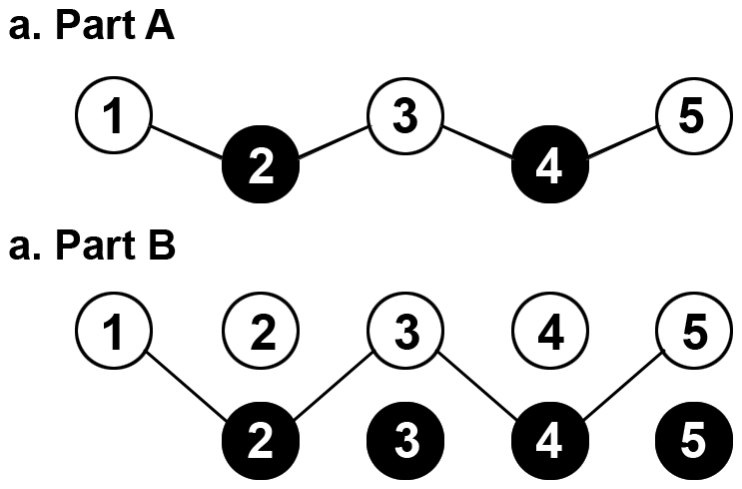
Trail Making Test-Black & White. The TMT-B&W Part A was similar to the TMT Part A, with an exception of all odd-numbers are within a white circle, and all even-numbers a black circle. The TMT-B&W Part B, each number is presented both in a white circle and a black circle, except the first encircled number is only written once.

The TMT-B&W retains the same psychometric properties as the TMT, but relies on the use of encircled numbers with black and white backgrounds instead of an English alphabet letters. TMT-B&W consists of two subsets, TMT-B&W part A and TMT-B&W part B (TMT-B&W-A, TMT-B&W-B). The part A consists of 25 circled numbers (1–25), with even numbers in a black circle and odd numbers in a white circle (See Figure 2–a). The part B displays all numbers (2–25) twice -except 1, which is presented only one time in a white circle-, each corresponding number encompassed in both a black and white circle. The trace of TMT-B&W matches up with that of the TMT.

In TMT-B&W-A, the subject is instructed to draw a line to connect the circles in ascending order. TMT B&W-B consists of double the stimuli compared with the TMT B&W-A with two sets of the 25 numbers in each color (black and white). In TMT-B&W-B, the participant is required to connect the numbers in consecutive order alternating between the two color sets. A maximum time of five minutes was allowed. To make sure each subject fully understood how to do the task, a practice trial session was given to them before the test trial.

The time taken to complete the task was measured in seconds. Errors were also counted, but did not serve as an analysis factor.

### Other Neuropsychological Assessments

A series of neuropsychological assessment tools were used to examine a wide variety of cognitive functions: attention, verbal and visual memory, visuospatial ability, frontal lobe functions, and language ability (See Table 2).

**Table 2:** Neuropsychological assessments.

Order	List of neuropsychological assessments
1	Mini Mental Status Examination
2	Verbal Learning Test
3	Rey Complex Figure Test
4	Digit span: forward & backward
5	Stroop test
6	Controlled Oral Word Association Test
7	The Boston Naming Test

### Data Analysis

A one-way analysis of covariance (ANCOVA) test was performed to assess differences in time-to-completion on the TMT and TMT-B&W among the groups with the age entered as a covariate.

In order to determine whether the TMT-B&W can be applied to undereducated participants, the test results of participants with high levels (+6years) and low levels (0–5years) were separately calculated. A Correlation analysis was carried out to identify the construct validity of the TMT-B&W and the relations with the TMT-B&W and other neuropsychological tests including the TMT. All analyses were done with the PASW statistics, version 18.0. Statistical significance level was set to P<0.05.

## Results

### 1. Overall Difference in the Time to Completion between the TMT and TMT-B&W

The results of time-to-completion in both the TMT Part A and B revealed that there were statistically significant differences among the groups (*F*_(2,46)_ = 2.64, *F*_(2,46)_ = 6.43, *p*<0.05) (See Table 3). The post hoc comparisons showed that performance of the NC and MCI group were significantly different from the AD group, while there was no significant gap between the NC and the MCI groups.

**Table 3:** Summary Data for participant Groups on TMT.

		TMT-A		TMT-B	
Group	N	M	SD	M	SD
NC	19	47.65*	2.56	106.12*	9.35
MCI	20	47.64†	2.35	125.97†	8.57
AD	11	56.14* †	3.25	162.56* †	11.84

Abbreviations; NC, Normal Controls; MCI, Mild Cognitive Impairment; AD, Alzheimer’s disease; TMT-A, Trail Making Test Part A; TMT-B, Trail Making Test Part B.

Note.

*p<0.05 for NC versus AD.

† p<0.05 for MCI versus AD.

Among the groups, there were significant differences in regard to the time-to-completion on the TMT-B&W-B (*F*_(2,85)_ = 15.83, *p*<0.05), however, not on Part A (See Table 4). The post hoc comparison suggested that the performance of the three groups were considerably different from one another.

**Table 4:** Summary Data for participant Groups on TMT-B&W.

		TMT-B&W-A		TMT-B&W-B	
Group	N	M	SD	M	SD
NC	28	53.73	5.06	126.72* †	9.05
MCI	42	70.16	4.40	154.52* ‡	7.11
AD	19	70.65	6.55	206.19† ‡	10.58

Abbreviations; N, number of participants; M, Mean; SD, Standard deviation; NC, Normal Controls; MCI, Mild Cognitive Impairment; AD, Alzheimer’s disease; TMT-B&W-A, Trail Making Test Black&White Part A; TMT-B&W-B, Trail Making Test Black&White Part B.

Note.

*p<0.05 for NC versus MCI.

† p<0.05 for NC versus AD.

‡ p<0.05 for MCI versus AD.

### 2. Comparison of the Completion-ratio on the TMT-B&W and the TMT

Out of the total of 138 subjects, 88 were designated in the high education group(≥6 years). For the TMT, only 76% of the NC; about 60% of the MCI; and about the 37% with AD completed the tasks. Overall, the completion rate of the TMT-B&W was much higher than that of the TMT. Even participants with cognitive impairments complete the task. Among the 25 participants in the NC group, 24(96%) completed the task; of the total 33(MCI), 29(about 88%) subjects finished TMT-B&W amongst the 30 AD patients, as many as 18(60%) had successfully completed the TMT-B&W.

The observed phenomenon was even more drastic for participants with low education. Participants with low education (<6 years), including NC group, all failed to complete the TMT, while the TMT-B&W was completed by 4 out of 6 participants(about 67%) in the NC group. Among 22 MCI, 13(about 59%) subjects finished the task. As for 22 AD patients who had seen their cognition deteriorate, only one subject finished the TMT-B&W (about 5%). The completion rate of the TMT and the TMT-B&W are presented in Table 5.

**Table 5:** Ratio of completing TMT and TMT-B&W.

		Ratio of completion (%)	
Education	Group	TMT	TMT-B&W
≥6	NC	76	96
	MCI	60	87
	AD	36	60
<6	NC	0	67
	MCI	0	59
	AD	0	4

Abbreviations; NC, Normal Controls; MCI, Mild Cognitive Impairment; AD, Alzheimer’s disease; TMT, Trail Making Test; TMT-B&W, Trail Making Test Black&White.

### 3. The Analysis of the TMT-B&W According to Education Group

When the participants were divided into two groups(lower and higher education), the results of participants with higher education suggested that only the time-to-completion in the TMT-B&W-B was significantly different among the groups(NC, MCI and AD) (*F*_(2,67)_ = 44.14, *p*<0.05) (See Table 6). The post hoc comparison revealed all three diagnostic groups differed considerably in regards to the time-to-completion on TMT-B&W -B.

**Table 6:** Summary Data on TMT-B&W for Participant Groups with over 6 years of education.

		TMT-B&W-A		TMT-B&W-B	
Group	N	M	SD	M	SD
NC	24	47.67	4.80	108.13* †	6.90
MCI	29	58.29	4.17	133.08* ‡	5.95
AD	18	65.80	5.35	204.01† ‡	7.64

Abbreviations; N, number of participants; M, Mean; SD, Standard deviation; NC, Normal Controls; MCI, Mild Cognitive Impairment; AD, Alzheimer’s disease; TMT-B&W-A, Trail Making Test Black&White Part A; TMT-B&W-B, Trail Making Test Black&White Part B.

Note.

*p<0.05 for NC versus MCI.

† p<0.05 for NC versus AD.

‡ p<0.05 for MCI versus AD.

### 4. Correlation of the TMT-B&W with the Other Tasks

To identify the validity of TMT-B&W, the concurrent validity was examined by comparing the correlation coefficients of the TMT-B&W with the value of the TMT, CDR (SOB), MMSE, and other neuropsychological tests respectively (See Table 7).

**Table 7:** Correlation between TMT-B&W, TMT and cognitive measures.

	TMT-A	TMT-B	TMT-B&W-A	TMT-B&W-B
TMT-A	1	0.59*	0.75*	0.54*
TMT-B	0.59*	1	0.45	0.68*
TMT-B&W-A	0.75*	0.45	1	0.72*
TMT-B&W-B	0.54*	0.68*	0.72*	1
CDR SOB	0.52*	0.49	0.41	0.65*
MMSE	−0.38	−0.49	−0.63*	−0.57*
DSF	−0.29	−0.22	−0.19	−0.12
DSB	−0.26	−0.33	−0.56*	−0.36
BNT	−0.44	−0.42	−0.49	−0.60*
VLT-I	−0.44	−0.52*	−0.46	−0.60*
VLT-D	−0.35	−0.55*	−0.35	−0.64*
VLT-R	−0.38	−0.36	−0.30	−0.51*
RCFT Copy	−0.37	−0.49	−0.67*	−0.54*
RCFT-I	−0.26	−0.38	−0.48	−0.54*
RCFT-D	−0.32	−0.40	−0.43	−0.50*
RCFT-R	−0.25	−0.30	−0.30	−0.41
COWAT-S	−0.44	−0.54*	−0.47	−0.65*
COWAT-P	−0.46	−0.42	−0.31	−0.45
Stroop test-W	−0.16	−0.22	−0.40	−0.30
Stroop test-C	−0.51*	−0.60*	−0.51*	−0.66*

Abbreviations; TMT, Trail Making Test; TMT-A, Trail Making Test Part A; TMT-B, Trail Making Test Part B; TMT-B&W, Trail Making Test Black&White TMT-B&W-A, Trail Making Test Black&White Part A; TMT-B&W-B, Trail Making Test Black&White Part B; CDR SOB, Clinical Dementia Rating Some Of Box; MMSE, Mini Mental Examination; DSF, Digit Span Forward; DSB, Digit Span Backward; BNT, Boston Naming Test; VLT-I, Verbal Learning Test-Immediate recall test; VLT-D, Verbal Learning Test-20-minute delayed recall test; VLT-R, Verbal Learning Test-Recognition test; RCFT copy, Rey Complex Figure Test copy; RCFT-I, Rey Complex Figure Test-Immediate recall test; RCFT-D, Rey Complex Figure Test-20-minute delayed recall test; RCFT-R, Rey Complex Figure Test-Recognition test, COWAT-S, Controlled Oral Word Association Test-Semantic word fluency; COWAT-P, Controlled Oral Word Association Test-Phonemic word fluency, Stroop test-W, Stroop test-Word reading; Stroop test-C, Stroop test-Color reading; *p<.01.

The results showed that the TMT-B&W significantly correlated with the TMT. Moreover, Part B of the TMT-B&W showed a high correlation with the Controlled Oral Word Association Test-Semantic word fluency (COWAT-S); Stroop test color reading (Stroop test-C). Part B of the TMT showed a correlation with those two tests, as well. Part B of the TMT also showed a correlation with the CDR SOB, MMSE, Boston Naming Test (BNT), Verbal Learning Test (VLT)-Immediate recall test, VLT-20-minute delayed recall test, VLT-Recognition test, Rey Complex Figure Test (RCFT) copy, RCFT-Immediate recall test, and the RCFT-20-minute delayed recall test.

## Discussion

The TMT is one of the most useful neuropsychological tests. Many clinicians and neuropsychologists in non-native English speaking countries cannot effectively administer the TMT because of its limited utility in cross-cultural settings. In Korea, it has been hard to get test results from the TMT [30]. This study showed there were many patients who were not familiar with the English alphabet and thus did not even attempt the TMT. Even those who tried to conduct the TMT quit halfway through the test. Thus alternative forms of the TMT and other modifications are required to be introduced with an aim to improve diagnostic utility in the evaluation of neurobehavioral disorders.

This study aimed to identify a new type of TMT that maintains the diagnostic utilities of the TMT. First, we tried to identify if Korean participants willingly performed the TMT-B&W and then we were dedicated to verify if the TMT-B&W served as an adequate substitute to the original. If so is there a possibility that the TMT-B&W is applicable in a clinical setting.

As the results show, the completion rate of the TMT-B&W was much higher than that of the TMT. In other words, a significantly higher number of participants failed to complete the TMT. It could be explained in several ways: one is that a number of patients failed to finish the TMT; another is some of the subjects refused to start the task in the first place and others gave up halfway through the task. In particular this study revealed that 24% of participants with high education (NC) could not complete the task. The completion rate was 60% for MCI, and 36% for AD. Those numbers demonstrate well that the TMT has limited applicability in cross-cultural settings. The shortcoming stood out when the test was administered to patients with low education levels. Regardless of cognitive impairment levels, participants with low education (including NC) all failed to accomplish the TMT: some of them stubbornly resisted starting the test; others gave up from the beginning. Ironically the TMT, that was originally devised to measure the level of cognitive impairment, seemed to be inapplicable to cognitively impaired patients.

Interestingly the overall failure rate of the TMT-B&W was low. Even for those with low education and low a level of cognitive abilities, the failure rate of the TMT-B&W was much lower than that of the TMT. In participants with high education, among the 25 participants in the NC group, 1(4%) failed the task; of the total 33 MCI, 4(about 12%) subjects failed TMT-B&W amongst the 30 AD patients, as many as 12(40%) had uncompleted the TMT-B&W. The major reason for the failure in TMT-B&W was not because of participants’ unwillingness but because of time limitations. The number of participants who were unwilling to complete the test or gave up was 1 subject in MCI, 3 subjects in AD. One participant in NC, 3 in MCI and 9 in AD could not complete the task within the time limit. The TMT-B&W performance rate of those who finished up the task is evident in Table 8. If we were to also include the participants who over timed the task, the TMT-B&W performance rate would go up significantly. That means, if the task was given with a time extension or without a time limit, the performance rate of TMT-B&W would rise significantly. We would conclude that TMT-B&W is applicable for patients with low education and low level cognitive functions.

**Table 8:** Ratio of performing the TMT-B&W.

Education	Group	TMT-B&W
≥6	NC	96
	MCI	90
	AD	70
<6	NC	83
	MCI	100
	AD	86

Abbreviations; NC, Normal Controls; MCI, Mild Cognitive Impairment; AD, Alzheimer’s disease; TMT-B&W, Trail Making Test Black&White.

In a comparison to time consumed on the TMT and TMT-B&W among those with high education, the level of cognitive impairment contributed to an overall time-to-completion gap on both TMT -A and B. However, in the case of TMT-B&W, only Part B showed a significant influence on time-to-completion. However, there was no considerable time difference between the NC and the MCI on the TMT-B within the group comparison, and the significant difference existed only in the NC versus the AD groups and the MCI versus the AD groups. But the results showed that there were significant time-to-completion differences among the groups on the TMT-B&W-B. That indicates the TMT-B&W -B can be a more useful tool to determine cognitive impairments of patients. Therefore we may conclude that the TMT-B&W can play a more effective role in distinguishing the level of cognition for each patient as compared to the TMT, especially for non-English speakers.

But the gap in time-to-completion in TMT-B&W Part A and B among groups was not noticeably different for those with low education. That was because few of them managed to finish the task of the TMT-B&W within the time limit. Thus, as mentioned earlier, it is expected that when patients are given enough time to complete the task or allowed to perform the task without time limit, then more patients will be likely to complete the TMT-B&W. One other possible reason for no difference in lower educated participants is that the number of subjects with low education was not sufficient in this study. The small sample size in each group did not demonstrate significant differences in time-to-completion.

Like the TMT, TMT-B&W was found to be highly comparable with other neuropsychological tests, including frontal executive function tests. It is fair to say that the TMT-B&W serves a similar diagnostic function as the TMT does. In particular the results from Part B of TMT-B&W and the TMT-B showed a high correlation with COWAT-S and Stroop test-C demonstrating that TMT-B&W is highly likely viewed as a sensitive tool to estimate the frontal lobe functions. We may conclude that the TMT and TMT B&W are essentially equal in their diagnostic value.

Based on the results of this study, it is more desirable to use the TMT-B&W package to assess the level of cognitive impairment in non-native English speaking countries. The TMT-B&W serves as an adequate substitute to the TMT, and is even more useful in identifying differences in time completion between certain groups. Furthermore, it is aimed at assessing cognitive functionality of particularly those who don’t have knowledge of the English alphabet or undereducated. For further study, the extension of time limit and an increased number of subjects are recommended in order to identify a possibility of application of the TMT-B&W to poorly educated patients. We also recommend that the applicability of TMT-B&W be analyzed in other countries (i.e. non-native English speaking ones); and a comparison study of TMT B&W and the TMT in native English speaking countries carried out.
